# Optimization of mouse kidney digestion protocols for single-cell applications

**DOI:** 10.1152/physiolgenomics.00002.2024

**Published:** 2024-03-25

**Authors:** Jake N. Robertson, Henry Diep, Alexander R. Pinto, Christopher G. Sobey, Grant R. Drummond, Antony Vinh, Maria Jelinic

**Affiliations:** ^1^Centre for Cardiovascular Biology and Disease Research, Department of Microbiology, Anatomy Physiology and Pharmacology, School of Agriculture, Biomedicine and Environment, La Trobe University, Bundoora, Victoria, Australia; ^2^Baker Heart and Diabetes Research Institute, Melbourne, Victoria, Australia

**Keywords:** flow cytometry, renal, single-cell RNA sequencing, tissue dissociation

## Abstract

Single-cell technologies such as flow cytometry and single-cell RNA sequencing have allowed for comprehensive characterization of the kidney cellulome. However, there is a disparity in the various protocols for preparing kidney single-cell suspensions. We aimed to address this limitation by characterizing kidney cellular heterogeneity using three previously published single-cell preparation protocols. Single-cell suspensions were prepared from male and female C57BL/6 kidneys using the following kidney tissue dissociation protocols: a scRNAseq protocol (*P1*), a multi-tissue digestion kit from Miltenyi Biotec (*P2*), and a protocol established in our laboratory (*P3*). Following dissociation, flow cytometry was used to identify known major cell types including leukocytes (myeloid and lymphoid), vascular cells (smooth muscle and endothelial), nephron epithelial cells (intercalating, principal, proximal, and distal tubule cells), podocytes, and fibroblasts. Of the protocols tested, *P2* yielded significantly less leukocytes and type B intercalating cells compared with the other techniques. *P1* and *P3* produced similar yields for most cell types; however, endothelial and myeloid-derived cells were significantly enriched using *P1*. Significant sex differences were detected in only two cell types: granulocytes (increased in males) and smooth muscle cells (increased in females). Future single-cell studies that aim to enrich specific kidney cell types may benefit from this comparative analysis.

**NEW & NOTEWORTHY** This study is the first to evaluate published single-cell suspension preparation protocols and their ability to produce high-quality cellular yields from the mouse kidney. Three single-cell digestion protocols were compared and each produced significant differences in kidney cellular heterogeneity. These findings highlight the importance of the digestion protocol when using single-cell technologies. This study may help future single-cell science research by guiding researchers to choose protocols that enrich certain cell types of interest.

## Graphical abstract

**Figure:**
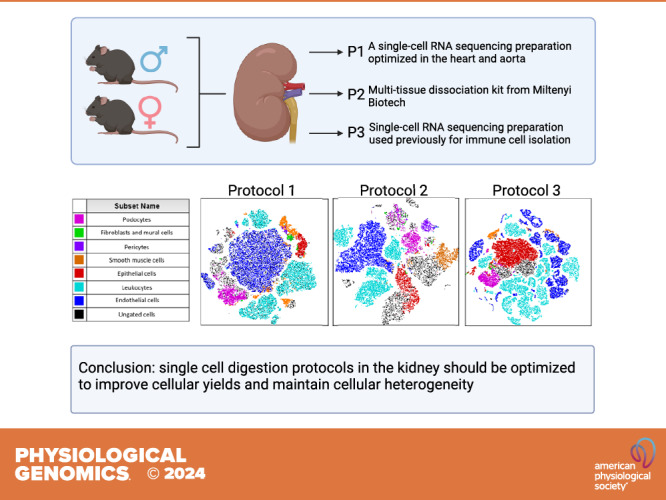

## INTRODUCTION

The prevalence of chronic kidney disease (CKD) is rapidly increasing worldwide and poses a major health burden for many countries (1). Although global age-standardized mortality for leading causes of death has steadily declined over the past 30 years, the age-standardized mortality for CKD has remained relatively stable (2). This highlights that there are unmet needs and potential barriers to CKD research. One of these unmet needs is a poor understanding of the cellular changes that occur during the pathogenesis of kidney disease (3). Single-cell approaches (such as flow cytometry and single-cell RNA sequencing; scRNAseq) have enabled in-depth characterization of kidney cellular composition. However, a major factor that influences the information derived from single-cell datasets is the approach used to prepare single-cell suspensions from whole tissues.

The kidney, like most organs, has heterogeneous tissues comprised of many cell types. Previous single-cell studies report that the kidneys are primarily comprised of epithelial cells, the majority of which are proximal tubule cells (4–7). Additional epithelial subtypes include cells of the nephron (loop of Henle cells, distal tubule cells, principal cells, A and B-intercalating cells) (8–10). Podocytes and parietal cells are two highly specialized types of epithelial cells that assist in maintaining filtration in the glomerulus (11, 12). Other major cell types in addition to epithelial cells include: cells of the renal vasculature (primarily endothelial and vascular smooth muscle cells) (13, 14); leukocytes, which eliminate foreign pathogens and contribute to tissue healing (15); and fibroblasts, the main producers of extracellular matrix proteins (16). All these major cell types can become dysfunctional or damaged during the progression of CKD. A clear understanding of the cell populations (and subpopulations) that exist in healthy and diseased kidneys will help to provide new insights into the cellular mechanisms of CKD.

An important factor in single-cell approaches is the quality of the single-cell suspension used. High cell viability and broad diversity of live cells will improve the quality of the data produced (17). Currently, there are no data that evaluate published single-cell suspension preparation protocols and their ability to produce high-quality cellular yields in the kidney. One consideration is the potential for single-cell suspension preparation protocols to enrich for certain cell types. In “omics” approaches such as scRNAseq, the enriched larger cell populations can prevent detailed analyses of smaller populations (18). Investigating the efficacy of current single-cell preparation protocols may dramatically improve cellular yields or help to enrich for certain cell types of interest.

There are many variations in single-cell suspension preparation protocols for single-cell applications that likely depend on the cell types of interest and may not consider the impact of the protocol on kidney cellular heterogeneity. One study investigating mouse kidney tissue, used a digestion protocol for scRNAseq where the majority of cells analyzed were endothelial cells and macrophages (19). Notably, this protocol has also been used previously in studies focused on heart tissue for scRNAseq. The Miltenyi Biotec multi-tissue dissociation kit is one dissociation method most widely used for kidney scRNAseq and flow cytometry experiments (4–6, 20). One of the first studies to use scRNAseq in the kidney used the Miltenyi multi-tissue digestion kit and was able to gain an understanding of kidney heterogeneity enough to identify a novel cell type in the collecting duct (4). Notably, with this being one of the first studies of kidney heterogeneity, the impact of the digestion protocol on the cellular populations is relatively unknown. In addition to the Miltenyi Biotec protocol, our previous publications have utilized a cell preparation protocol to specifically study renal leukocytes (21, 22). Notably, other major kidney cell types have not been enumerated using this approach. Therefore, to optimize renal sample digestion for single-cell studies, we aimed to empirically compare the performance of these three single-cell suspension preparation protocols in the mouse kidney. We also compared the single-cell suspension preparations between males and females, to determine whether the different protocols performed similarly in both sexes.

## MATERIALS AND METHODS

### Animals

All animal experiments were approved by La Trobe University Ethics Committee (AEC No. 16-93). Male and female C57BL/6 mice aged 10- to 12-wk old were used in this study (*n* = 8 for each sex). Mice were kept in 12-h light/dark cycle with ad libitum access to normal chow diet (Specialty Feeds Perth, Western Australia) and drinking water in the La Trobe Animal Research & Training Facility (LARTF). Mice were housed in same-sex groups of four in individually ventilated cages (Tecniplast, Rydalmere, Australia). At end point, mice were killed by CO_2_ asphyxiation and perfused with phosphate buffered saline (PBS) through the left ventricle of the heart for 5 min. Both kidneys were removed, decapsulated, and halved using a scalpel. Each half was weighed and three were randomly allocated to one of the three single-cell suspension protocols described below in *Protocols*. The fourth portion of kidney for each digestion protocol was frozen for histological analyses.

### Preliminary Optimization Steps

Preliminary studies on a separate cohort of six male mice compared mechanical dissociation with GentleMACS Dissociator (Programs: Liver 1 and 2; Miltenyi Biotec, North Rhine-Westphalia, Germany) to mincing tissues manually with dissecting scissors. Tissues that underwent GentleMACS dissociation were pre-cut into ∼2 mm cubes using dissecting scissors to assist with tissue mincing. Dissociation was performed for ∼5 min. Preliminary studies also tested whether debris clearance compromised cellular heterogeneity in single-cell suspensions (using kidneys from an additional 5 female C57BL/6 mice). Debris clearance was performed by centrifugation of cells at 200 *g* at 4°C for 15 min with the brakes off.

### Protocols

#### Protocol 1.

For *protocol 1* (*P1*), kidney tissue was cut into 1–2 mm cubes using dissecting scissors (performed for ∼1 min) and placed in an enzyme mixture of collagenase IV (2 mg/mL; Sigma-Aldrich, MA), dispase II (200 µg/mL; Sigma-Aldrich, MA), and DNase I (1 µg/mL; Roche, Victoria, Australia). Samples were then incubated in a 37°C water bath for 45 min and triturated 15 times every 15 min using a 1,000 µL pipette. Following incubation, the sample was passed through a 70-µm cell strainer and washed with PBS. Samples were then centrifuged at 300 *g* for 5 min at 4°C. The supernatant was discarded, and the pellet was resuspended in PBS and passed through 40-µm cell strainer. Samples were centrifuged at 300 *g* for 5 min at 4°C and resuspended again in PBS. One notable deviation was to remove an initial 200 *g* centrifugation for 15 min at 4°C (debris clearance), as the preliminary results displayed this reduced epithelial cell yield.

#### Protocol 2.

*Protocol 2* (*P2*) used the commercially available multi-tissue dissociation kit 2 (130-110-203; Miltenyi Biotec) with minor variations. Kidney tissue was finely minced with dissecting scissors in the prepared digestion enzyme solution (as per manufacturer’s instructions). Kidney tissue was then incubated in Incu-shaker (Benchmark scientific, NJ) for 30 min at 37°C. Cells were washed with PBS and filtered through 70-µm cell strainer before being centrifuged at 300 *g* for 5 min at 4°C. The supernatant was discarded and the pellet was resuspended in PBS and passed through 40-µm cell strainer. Samples were centrifuged at 300 *g* for 5 min at 4°C and resuspended again in PBS. One deviation from manufacturer recommendations was removing the use of the gentleMACS dissociator as this mostly had a negative impact on cell populations compared with mincing with dissection scissors.

#### Protocol 3.

For *protocol 3* (*P3*), kidney tissue was finely minced with dissecting scissors (performed for ∼2–3 min) in a digestion solution of collagenase XI (0.15 mg/mL; Sigma-Aldrich, MA), hyaluronidase (30 µg/mL; Sigma-Aldrich, MA), collagenase I-S (2 mg/mL; Sigma-Aldrich, MA), and DNase (1 µg/mL; Roche, Victoria, Australia) as previously described (21, 22). Samples were then incubated and agitated in an Incu-shaker for 60 min at 37°C. Cells were washed with PBS and filtered through 70-µm cell strainer before being centrifuged at 300 *g* for 5 min at 4°C. The supernatant was discarded and the pellet was resuspended in PBS and passed through 40-µm cell strainer. Samples were centrifuged at 300 *g* for 5 min at 4°C and resuspended again in PBS.

### Cell Staining and Flow Cytometry

**Figure 1.**
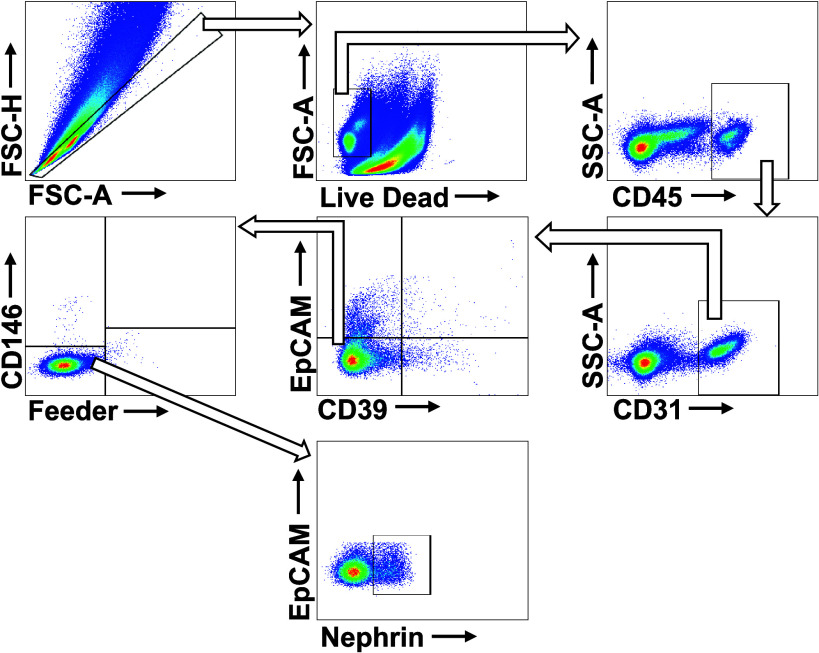
Representative image of the gating strategy of general cell types of the kidney. Cells were labeled using the general kidney cell antibody panel. Multiplets and debris were removed by gating forward scatter area (FSC-A) against forward scatter height (FSC-H). Live cells were determined as those negative for aqua live/dead stain. Leukocytes were identified as the CD45^+^ population. The CD45^−^ population was then gated for CD31 to identify endothelial cells (CD31^+^). The CD31^−^ population was divided into quarters testing for smooth muscle (CD39^+^) and epithelial cell populations (epithelial cell adhesion molecule, EpCAM^+^). The feeder cell stain (fibroblasts and mural cells) and CD146 stain (pericytes) divided the EpCAM^−^/CD39^−^ population. Finally, podocytes (nephrin^+^) were gated for using the Feeder^−^/CD146^−^ population.

**Figure 2.**
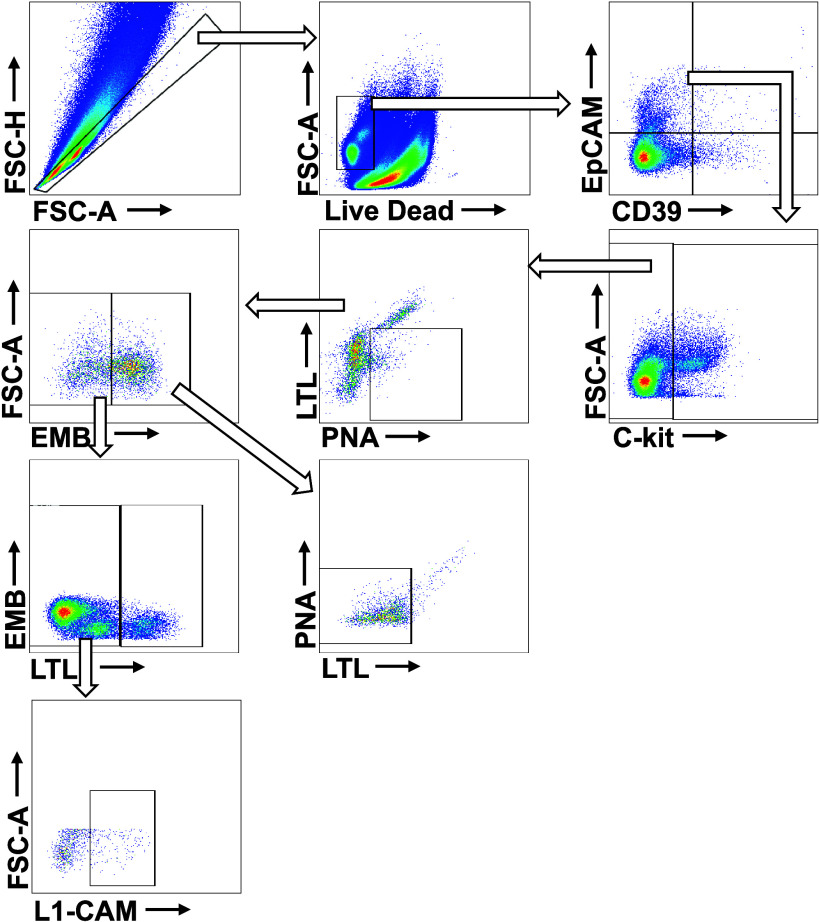
Representative image of the gating strategy for epithelial subtypes. Cells were labeled using the epithelial cell subtype antibody panel. Multiplets and debris were removed by gating forward scatter area (FSC-A) against forward scatter height (FSC-H). Using the epithelial cell adhesion molecule (EpCAM^+^) population, type A-intercalating cells were gated for using the c-kit stain. The c-kit^−^ population was then gated for type B-intercalating cells (peanut agglutinin lectin, PNA^+^/*Lotus tetragonoblus* lectin, LTL^−^). The PNA^−^ and LTL^+^ populations were then divided into the embigin (EMB^−^) (gated for proximal tubule cells; LTL^+^) and EMB^+^ (gated for distal tubule cells; LTL^−^/PNA^−^) populations. Finally, the LTL^−^ population gated for principal cells (L1 cell adhesion molecule, L1-CAM^+^).

**Figure 3.**
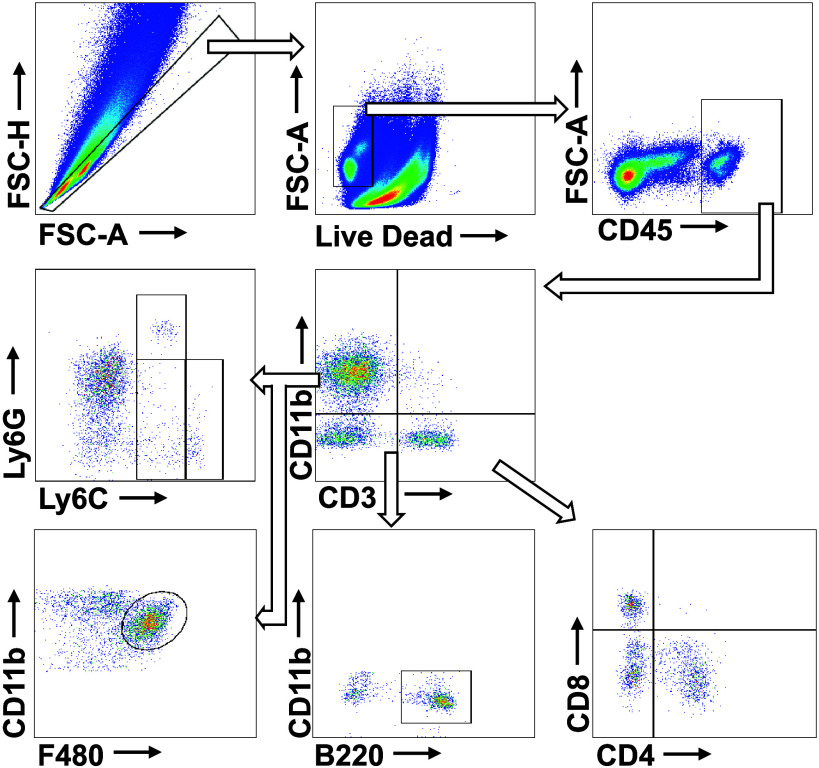
Represenative image of the gating strategy for leukocyte subtypes. Cells were labeled using the leukocyte subtype antibody panel. Multiplets and debris were removed by gating forward scatter area (FSC-A) against forward scatter height (FSC-H). Leukocytes (CD45^+^) were gated for myeloid (CD11b^+^) and T cell (CD3^+^) subtypes. CD11b^+^ population was further divided using Ly6C (monocytes), Ly6G (granulocytes) and F4/80 (macrophages). Low and high Ly6C^+^ populations were further divided into different populations (patrolling and proinflammatory monocytes). T cell subtypes were divided using CD4 (helper T cells) and CD8 (cytotoxic T cells) markers. B cells (B220^+^) were identified within the CD3^−^/CD11b^−^ population.

Cellular heterogeneity and abundance of the mouse kidneys was validated by labeling cells with fluorescent antibodies for flow cytometry. All single-cell suspensions were loaded onto 96-well U-bottomed plates and stained with aqua live/dead viability stain (1:1,000 dilution in PBS; Thermo Fisher, Victoria, Australia) at room temperature for 15 min. Samples were then washed with MACS buffer (PBS with 0.5% bovine serum albumin and 2 mM EDTA) and spun at 300 *g* for 5 min at 4°C and subsequently incubated with fluorophore-conjugated antibodies and stained for 20 min at room temperature using one of three distinct antibody panels (Tables 1, 2 and 3). Following antibody incubation, cells were washed with MACS buffer and spun at 300 *g* for 5 min at 4°C. Supernatant was discarded and cells were resuspended in MACS buffer. Samples were analyzed on a CytoFLEX analyzer (Beckman Coulter, Indianapolis, IN) flow cytometer using CytExpert software version 2.5 (Beckman Coulter) Postacquisition, the data were analyzed using FlowJo software v10.8.1 (BD Life Sciences) (29). For the full gating strategy, see Figs. 1, 2, and 3. Gate placement was confirmed through fluorescence minus one staining.

**Table 1.** General kidney cell panel

Target Cell Type	Clone	Antigen	Fluorophore	Supplier	Concentration	Reference
Endothelial cells	390	CD31	BV605	Biolegend	0.4 mg/mL	(23)
Pericytes	ME-9F1	CD146	BV650	Miltenyi	0.4 mg/mL	(24)
Fibroblasts and MCs	mEF-SK4	Feeder cells	PE	Miltenyi	0.2 mg/mL	(25)
Epithelial cells	G8.8	EpCAM	PerCP-Cy5.5	Biolegend	1 mg/mL	(25)
SMCs	Duha59	CD39	PE-Cy7	Biolegend	1 mg/mL	(26)
Podocytes	G-8	Nephrin	AF647	Bioss	0.2 mg/mL	(27)

Conjugated fluorescent antibodies for flow cytometry to identify major cell types of the kidney are shown. The antibody suppliers included BioLegend, Miltenyi Biotech (North Rhine-Westphalia, Germany), and Bioss Antibodies. EpCAM, epithelial cell adhesion molecule; MCs, mural cells; SMCs, smooth muscle cells.

**Table 2.** Epithelial cell subtype panel

Target Cell Type	Clone	Antigen	Fluorophore	Supplier	Concentration	Reference
PTCs	FL-1321	LTL	FITC	Vector	0.4 mg/mL	(8)
DCTCs	REA501	EMB	APC	Miltenyi	0.75 mg/mL	(8)
A-ICs	2B8	c-kit	Cy7	Biolegend	0.2 mg/mL	(10)
B-ICs	FL-1321	PNA	Cy5	Vector	0.4 mg/mL	(10)
Principal cells	555	L1-CAM	PE	Miltenyi	1 mg/mL	(10)
Epithelial cells	G8.8	EpCAM	PerCP-Cy5.5	Biolegend	0.2 mg/mL	(28)

Conjugated fluorescent antibodies for flow cytometry to identify epithelial cell subtypes of the kidney are shown. The antibody suppliers included Vector Biolabs (Malvern, PA), Miltenyi Biotech (North Rhine-Westphalia, Germany), and BioLegend. A-ICs, A-intercalating cells; B-ICs, B-intercalating cells; DCTCs, distal convoluted tubule cells; EMB, embigin; EpCAM, epithelial cell adhesion molecule; L1-CAM, L1 cell adhesion molecule; LTL, *Lotus tetragonoblus* lectin; PNA, peanut agglutinin lectin; PTCs, proximal tubule cells.

**Table 3.** Leukocyte subtype fluorescent antibody panel

Target Cell Type	Clone	Antigen	Fluorophore	Supplier	Concentration	Reference
Leukocytes	30-F11	CD45	AF700	BioLegend	1 mg/mL	(21)
T cells	145-2C11	CD3	APC	BioLegend	0.4 mg/mL	(21)
Myeloid cells	M1-70	CD11b	BV421	BioLegend	0.5 mg/mL	(21)
T helper/T regs	RM4-5	CD4	BV605	BioLegend	0.4 mg/mL	(21)
Cytotoxic T cells	53-6.7	CD8	PerCP-Cy5.5	BioLegend	0.2 mg/mL	(21)
Macrophages	BM8	F4/80	APC-Cy7	BioLegend	0.4 mg/mL	(21)
Granulocytes	1A8	Ly6G	PE-Cy7	BioLegend	0.2 mg/mL	(21)
Monocytes	HK1.4	Ly6C	FITC	BioLegend	0.2 mg/mL	(21)
B cells	RA3-6B2	B220	PE	BioLegend	0.2 mg/mL	(21)

Conjugated fluorescent antibodies for flow cytometry to identify immune cell subtypes of the kidney are shown. The antibody supplier was BioLegend. T regs, T regulatory cells.

### Digestion of Spleen Tissue

Further inquiry into the effect of single-cell digestion preparations on T cell populations was necessary after observing the results in the kidney. The spleen is an organ that is routinely digested without enzymes and thus can be used as a control for investigating the effect of different enzymes on cellular populations. This “undigested” protocol was completed by mincing spleen tissue and passing it through a 70-µm filter into a 50-mL falcon tube containing red blood cell lysis buffer (0.15 M of ammonium chloride, 0.025 M of potassium, and 1.2 × 10^−4^ M of EDTA). Samples were incubated in red blood cell lysis buffer for 10 min and then washed with PBS. Samples were then centrifuged under the same conditions and the supernatant was discarded. Subsequently, cells were resuspended in PBS. This digestion method was used in combination with *P1* and *P3* to investigate the validity of possible epitope cleavage seen in cytotoxic T cells and T helper cells using the *P1* single-cell digestion protocol.

### Immunofluorescence Staining

Kidneys were frozen in Tissue-Tek Optimal Cutting Temperature (OCT) compound (Sakura Finetek, CA) following tissue collection. Samples were cut into 10 µm sections using a cryostat (Leica Biosystems, Victoria, Australia) at −20°C and placed onto Superfrost Plus Adhesion Microscope Slides (Epredia, North Brabant, The Netherlands). Slides were then stored at −80°C until use. Initially, slides were acclimated to room temperature for 10 min and then fixed in 10% neutral buffered formalin solution for 10 min. Sections were then washed three times for 5 min on an Orbital shaker (Ratek, South Australia, Australia) in PBS with 0.05% Tween-20 (PBS-T; Sigma-Aldrich, MO). Kidney sections were then incubated at room temperature with Background Sniper (Biocare Medical, CA) for 10 min. The blocking solution was gently drained off the slide and sections were then incubated overnight at 4°C with rat monoclonal epithelial cell adhesion molecule (EpCAM) (G8.8) IgG_2a_ antibody (5 µg/mL, sc-53532, Santa Cruz Biotechnology, TX) in PBS-T containing 10% normal goat serum with 0.1% Triton-X (Sigma-Aldrich, MO) or in the antibody diluent alone as a negative control. Samples were then washed three times with PBS-T and incubated at room temperature with Alexa Fluorophore 594 conjugated goat anti-rat antibody (2 µg/mL, A11007, Invitrogen, MA) in PBS. The slides were then set in Vectashield antifade mounting medium with DAPI (Vector laboratories, CA). Images were captured using a BX53 epifluorescent microscope (Olympus Life Science Solutions, Tokyo, Japan).

### Statistical Analysis

All data are expressed as the means ± SE; *n* represents the number of animals per group. All statistical analyses were performed in GraphPad Prism 9 (GraphPad Software, San Diego, CA) using two-way ANOVAs with Šidák post hoc analysis. A level of *P* < 0.05 was considered statistically significant. The t-SNE plots were produced from data concatenated from two male and two female mice (*n* = 4).

## RESULTS

### Effect of Debris Clearance and Automated Mechanical Dissociation

**Figure 4.**
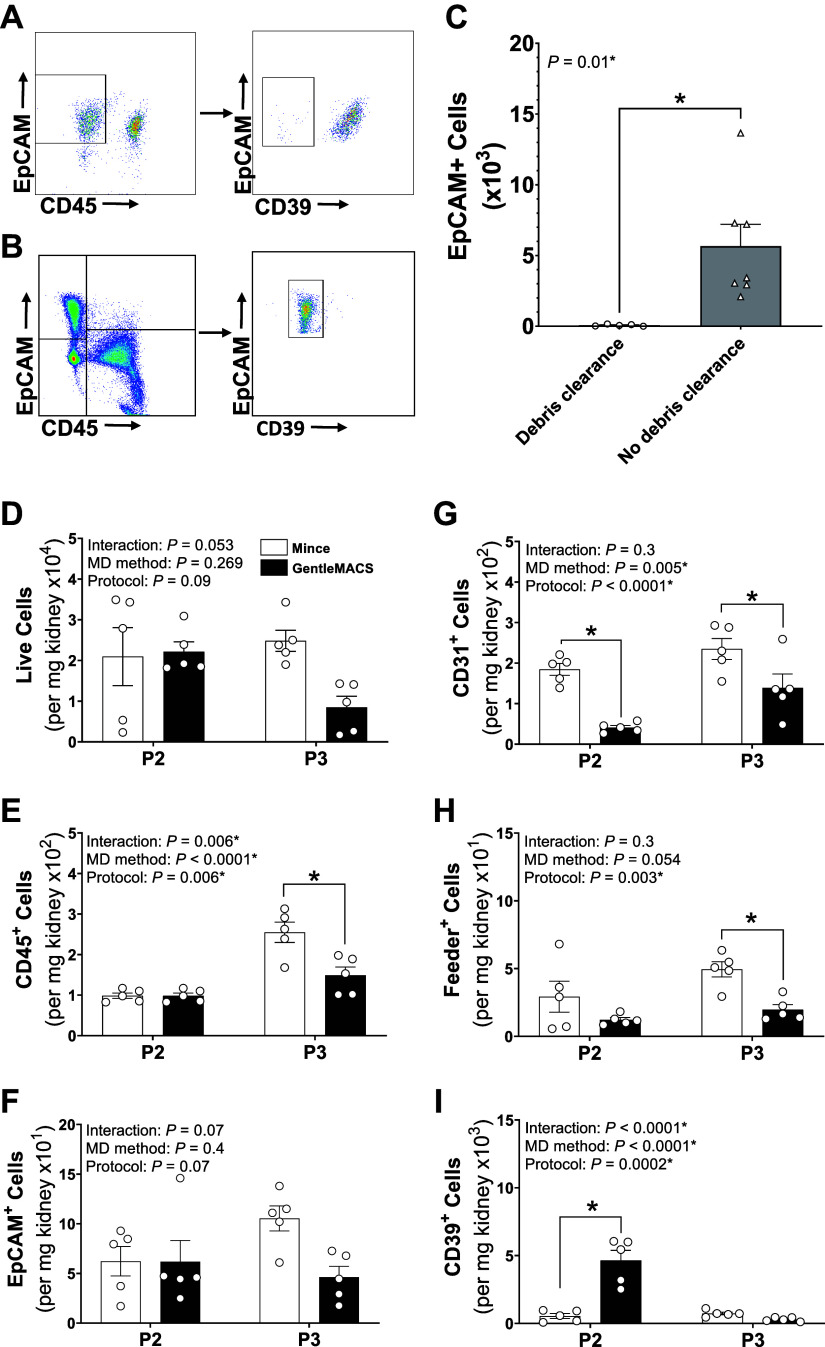
Debris clearance and GentleMACS dissociation reduced renal cell yields. *A−C*: representative flow cytometry plots of renal single-cell suspensions (*A* and *B*) and epithelial cell (epithelial cell adhesion molecule, EpCAM^+^) counts (*C*) from preparations with (○) and without (△) debris clearance. *D−I*: mean flow cytometric data of viable cells (*D*; aqua live/dead^−^), leukocytes (*E*; CD45^+^), epithelial cells (*F*; EpCAM^+^), endothelial cells (*G*; CD31^+^), fibroblasts, mural cells (*H*; Feeder^+^), and smooth muscle cells (*I*; CD39^+^) per mg of kidney tissue following automated gentleMACS (black bars) and manual mincing (white bars) of single-cell suspensions prepared using *protocol 2* (*P2*) and *protocol 3* (*P3*). *Significantly (*P* < 0.05) different compared with debris clearance (Student's *t* test) or gentleMACs dissociation (two-way ANOVA with Šidák post hoc analysis). Values are means ± SE, *n* = 5–7 animals.

Pilot experiments tested two steps of cell preparation: debris clearance and mechanical dissociation (Fig. 4, *A* and *B*). Debris clearance significantly reduced epithelial cell populations to a negligible level (Fig. 4, *A* and *C*). To investigate mechanical dissociation, the Miltenyi GentleMACS dissociator was compared with fine mincing with dissecting scissors. GentleMACS digestion did not affect total live yields, but reduced the yields of certain cell types analyzed (Fig. 4*D*). Specifically, GentleMACS reduced the yield of endothelial cells (*P2* and *P3*), immune cells (*P3* only), and fibroblasts (*P3* only) when compared with mincing (Fig. 4, *G* and *H*). GentleMACS had no effect on epithelial cell yields (Fig. 4*F*), but did improve vascular smooth muscle cell yields significantly (*P2* only; Fig. 4*I*). Overall, these pilot experiments identified that the debris clearance step and automated GentleMACS dissociation were detrimental to renal single-cell suspension preparations. Thus, these steps were not used in subsequent experiments.

### Differential Effects of Cell Preparation on General Kidney Cell Types

**Figure 5.**
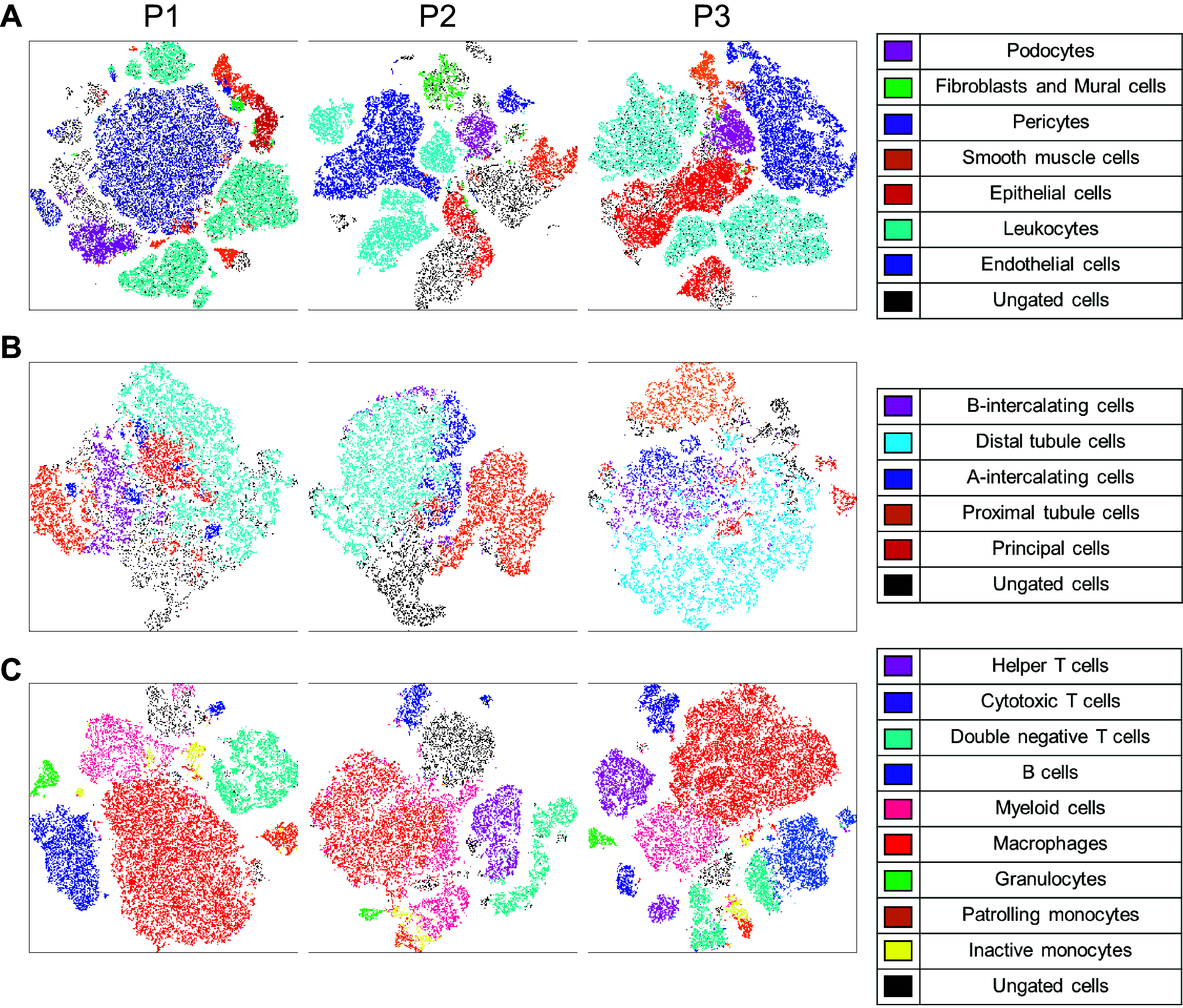
Global *t*-distributed stochastic neighbor embedding (tSNE) plots differed between each preparation protocol. *A−C*: representative t-SNE plots of general renal cell types (*A*), epithelial cell subtypes (*B*), and immune cell subtypes (*C*) in kidney samples processed using *protocols 1*, *2*, and *3* (*P1*, *P2*, and *P3*, respectively). Four kidney samples were concatenated from each protocol. Cell types are represented by dot color which is denoted in the corresponding legend (*right*).

**Figure 6.**
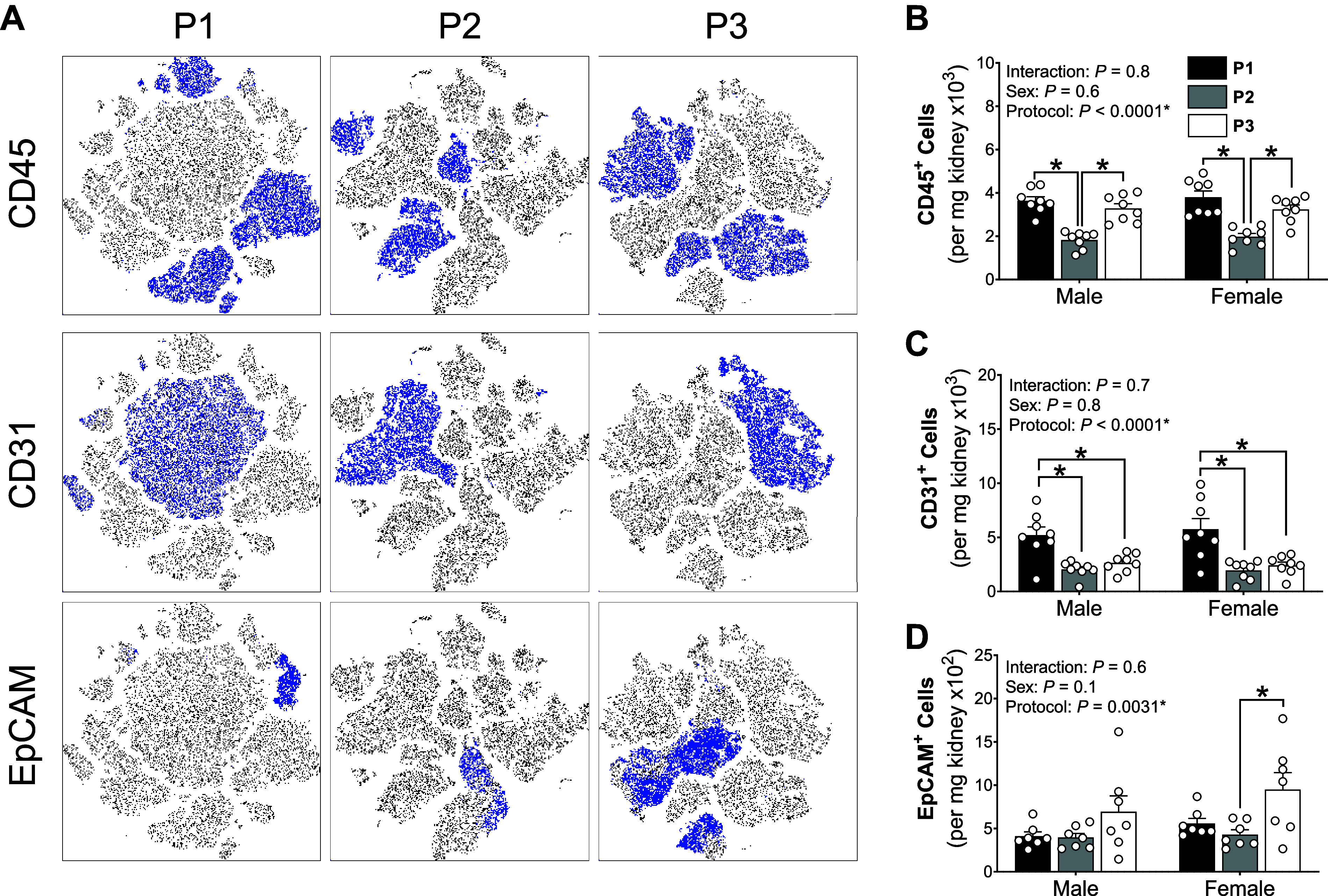
*Protocol 2* (*P2*) reduced leukocyte yields and *protocol 1* (*P1*) enriched endothelial cell yields. *A*: representative t-SNE plots of kidney samples process using *P1*, *P2*, and *protocol 3* (*P3*). Four kidney samples were concatenated from each protocol where blue dots depict cells that express the defined marker (*left*). *B−D*: total leukocyte (CD45^+^; *B*), endothelial cell (CD31^+^; *C*), and epithelial cell (EpCAM^+^; *D*) populations in male and female kidneys processed using *P1* (black bars), *P2* (gray bars), and *P3* (white bars) single-cell preparation protocols. *Significantly (*P* < 0.05) different between protocols (two-way ANOVA with Šidák post hoc analysis). Values are means ± SE, *n* = 7 or 8 animals.

Total viable cell counts were not affected by either digestion protocol or sex. However, there were notable differences in global *t*-distributed stochastic neighbor embedding (tSNE) plots between each of the three single-cell suspension preparation protocols (Fig. 5). Each cell type was then quantified individually to determine which populations were most affected by protocol type. There were no differences in pericyte (CD146^+^), fibroblast, and mural cell yields (Feeder^+^) between digestion protocols (Table 4). A significant difference between digestion protocols was observed in leukocyte, epithelial, and endothelial cell counts (Fig. 6*A*). Post hoc analyses revealed that leukocytes were significantly decreased in *P1* when compared with the other two methods (Fig. 6*B*). *P1* significantly enriched endothelial cells compared with the other two methods (Fig. 6*C*). *P3* significantly enriched for epithelial cells (in females only) compared with *P1* (Fig. 6*D*). Sex significantly affected only vascular smooth muscle cell yields; however, no post hoc comparisons were significantly different (Table 4). Notably, most live cells in this gating strategy were found to be unbound by fluorescent antibodies (referred to as “ungated cells” in Table 4).

**Table 4.** Proportion of major cell types in the kidney

Cell Type	*Protocol 1*		*Protocol 2*		*Protocol 3*	
	Male	Female	Male	Female	Male	Female
Pericytes	0.2 ± 0.05	0.2 ± 0.3	0.2 ± 0.1	0.1 ± 0.03	0.2 ± 0.04	0.2 ± 0.04
Fibroblasts	0.1 ± 0.03	0.2 ± 0.02	0.1 ± 0.04	0.2 ± 0.05	0.09 ± 0.03	0.1 ± 0.04
Podocytes	1.9 ± 0.5	3.2 ± 0.9	1.3 ± 0.5	1.2 ± 0.5	4.8 ± 2.2	7.0 ± 2.5*
Leukocytes	12.3 ± 2.1	13.1 ± 1.9	9.4 ± 1.6	10.7 ± 2.4	13.1 ± 2.5	10.4 ± 1.3
Epithelial cells	1.9 ± 0.7	3.8 ± 2.0	2.4 ± 0.7	4.1 ± 2.2	3.1% ± 1.0	4.8 ± 2.2
Endothelial cells	18.6 ± 3.2*	21.8 ± 3.8*	11.0 ± 1.9	11.3 ± 2.6	10.7 ± 1.8	8.1 ± 1.6
SMCs	0.8 ± 0.1	1.1 ± 0.2	0.5 ± 0.1	0.9 ± 0.2	0.6 ± 0.1	0.6 ± 0.1
Ungated cells	64.1 ± 5.5	56.6 ± 7.6	75 ± 4.1	71.5 ± 6.6	67.4 ± 5.6	68.8 ± 6.5

Values are means ± SE, *n* = 8 animals. Percentage of major renal cell types as a proportion of live cells in the kidney is shown. SMCs, smooth muscle cells.

*S ignificantly (*P* < 0.05) different between protocols (two-way ANOVA with Šidák post hoc analysis).

### Differential Effects of Cell Preparation on Renal Epithelial Cell Subtypes

**Figure 7.**
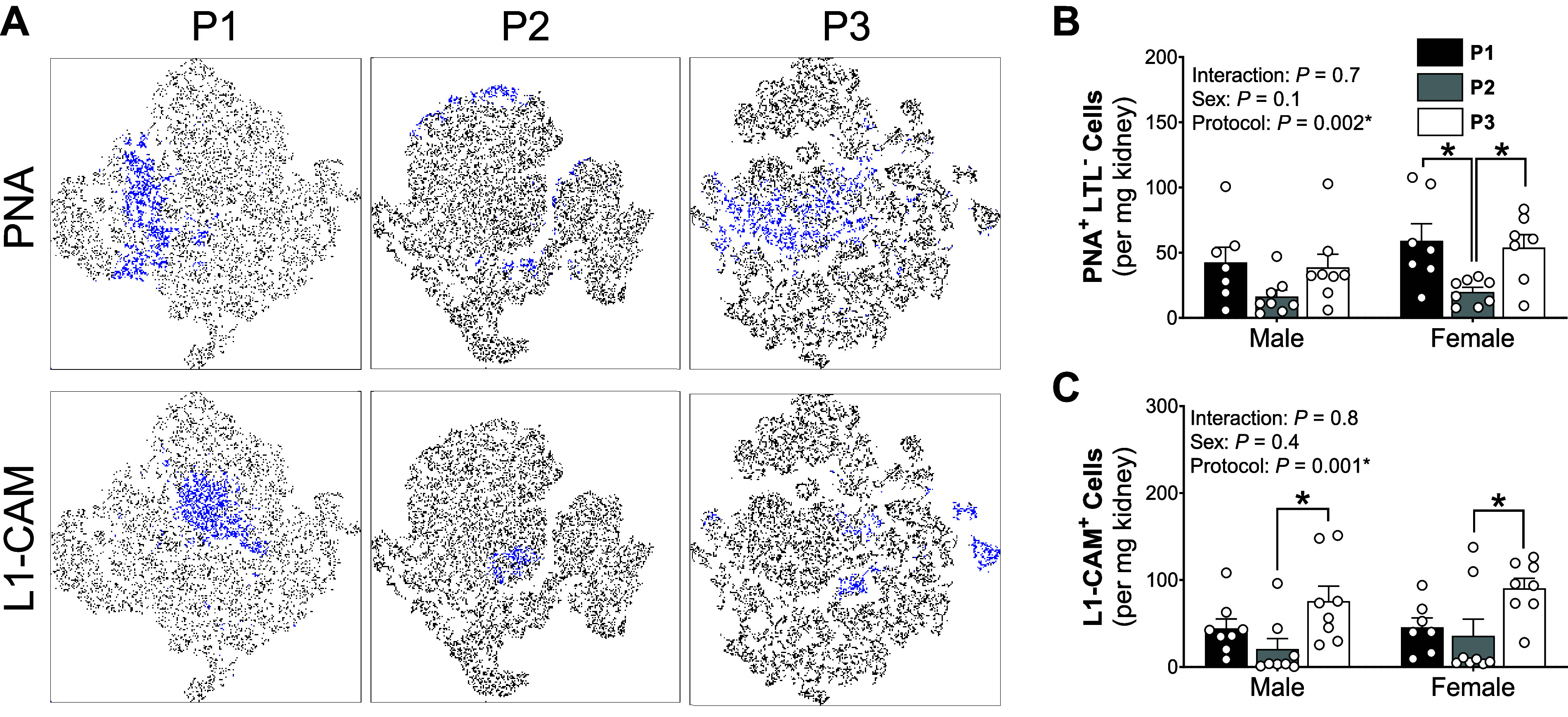
*Protocol 2* single-cell preparation protocol reduced epithelial subtype yields. *A*: representative t-SNE plots of kidney samples process using *protocols* *1*, *2*, and *3* (*P1*, *P2*, and *P3*, respectively) . Four kidney samples were concatenated from each protocol where blue dots depict cell that express the defined marker (*left*). *B* and C: total B-intercalating cells (peanut agglutinin lectin, PNA^+^/*Lotus tetragonoblus* lectin, LTL^−^; *B*) and principal cells (L cell adhesion molecule, L-CAM^+^; *C*) in male and female kidneys processed using *P1* (black bars), *P2* (gray bars), and *P3* (white bars) single-cell preparation protocols. *Significantly (*P* < 0.05) different between protocols (two-way ANOVA with Šidák post hoc analysis). Values are means ± SE, *n* = 7 or 8 animals.

No significant differences between protocols or sex were observed in type A-intercalating (c-kit^+^), distal tubule (embigin, EMB^+^) and proximal tubule cell types (*Lotus tetragonoblus* lectin, LTL^+^; Table 5). *P2* significantly reduced type B-intercalating cell yields (peanut agglutinin lectin, PNA^+^; Fig. 7, *A* and *B*). Post hoc analyses revealed this specifically occurred in samples from female mice. Principal cells (L1 cell adhesion molecule, L1-CAM^+^) were also significantly reduced in *P2* preparations compared with *P3* in both males and females (Fig. 7*C*).

**Table 5.** Proportion of epithelial cell subtypes

Cell Type	*Protocol 1*		*Protocol 2*		*Protocol 3*	
	Male	Female	Male	Female	Male	Female
A-intercalating cells	0.2 ± 0.05	0.2 ± 0.3	0.2 ± 0.1	0.1 ± 0.03	0.2 ± 0.04	0.2 ± 0.04
B-intercalating cells	0.1 ± 0.03	0.2 ± 0.02	0.1 ± 0.04	0.2 ± 0.05	0.09 ± 0.03	0.1 ± 0.04
DCTC	1.9 ± 0.5	3.2 ± 0.9	1.3 ± 0.5	1.2 ± 0.5	4.8 ± 2.2	7.0 ± 2.5
Principal cells	12.3 ± 2.1	13.1 ± 1.9	9.4 ± 1.6	10.7 ± 2.4	13.1 ± 2.5	10.4 ± 1.3
Proximal tubule cells	1.9 ± 0.7	3.8 ± 2.0	2.4 ± 0.7	4.1 ± 2.2	3.1 ± 1.0	4.8 ± 2.2

Values are means ± SE, *n* = 8 animals. Percentage of epithelial cell subtypes as a proportion of the total amount of epithelial cells in the kidney is shown. DCTCs, distal convoluted tubule cells.

### Differential Effects of Cell Preparation on Renal Immune Cell Subtypes

**Figure 8.**
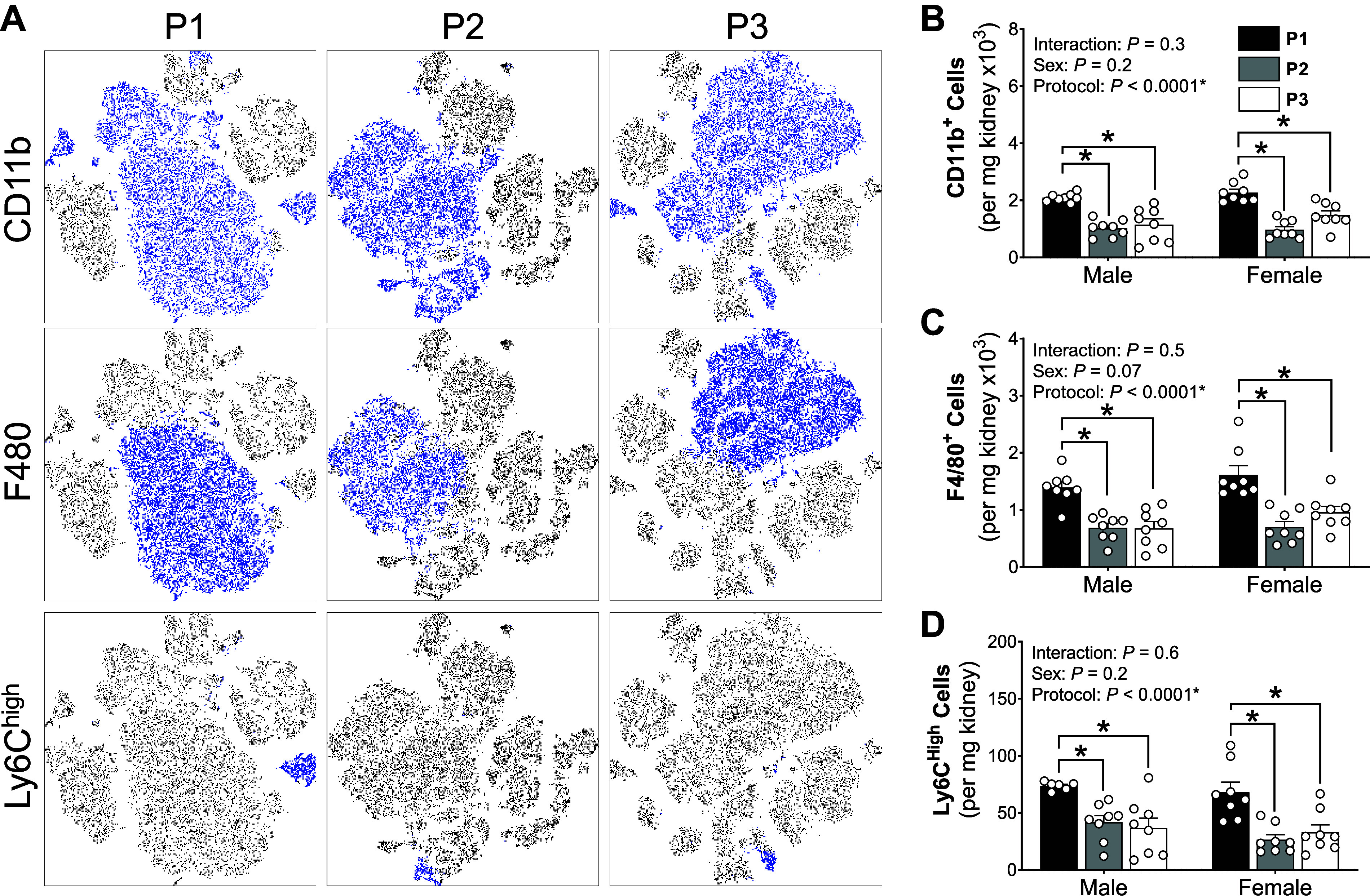
*Protocol 1* single-cell preparation protocol enriched myeloid-derived cell yields. *A*: representative t-SNE plots of kidney samples process using *protocols 1*,* 2*, and* 3* (*P1*, *P2*, and *P3*, respectively). Four kidney samples were concatenated from each protocol where blue dots depict cell that express the defined marker (*left*). *B*–*D*: total myeloid-derived cell (CD11b^+^; *B*), macrophage (F4/80^+^; *C*), and activated monocyte (Ly6C^hi^; *D*) populations in male and female kidneys processed using *P1* (black bars), *P2* (gray bars), and *P3* (white bars) single-cell preparation protocols. *Significantly (*P* < 0.05) different between protocols (two-way ANOVA with Šidák post hoc analysis). Values are means ± SE, *n* = 7 or 8 animals.

Myeloid-derived cells (CD11b^+^; Fig. 8, *A* and *B*; Table 6) were significantly greater in *P1* preparations compared with *P3* and *P2*. Similarly, macrophages (F4/80^+^) and proinflammatory monocytes (Ly6C^Hi^) were significantly greater in *P1* preparations compared with *P2* and *P3* in both sexes (Fig. 8, *C* and *D*, Table 6). Patrolling monocytes (Ly6C^Lo^) were also significantly greater in *P1* preparations compared with *P2*, but only in female mice (Table 6). Granulocyte yields (Ly6G^+^) were not affected by the single-cell suspension preparation protocols but were significantly greater in females, compared with males (Table 6).

**Table 6.** Proportion of immune cell subtypes

Cell Type	*Protocol 1*		*Protocol 2*		*Protocol 3*	
	Male	Female	Male	Female	Male	Female
Myeloid cells	66.0 ± 2.8*	66.7 ± 2.7*	56.3 ± 2.6	53.3 ± 3.0	48.1 ± 2.5	54.2 ± 2.7
Macrophages	65.7 ± 3.5	70.5 ± 3.6	66.5 ± 4.0	70.1 ± 2.9	62.3 ± 4.8	64.6 ± 4.3
Granulocytes	5.3 ± 1.3#	1.9 ± 0.2	6.9 ± 1.6#	3.1 ± 1.4	6.1 ± 2.2#	2.2 ± 0.3
Monocytes^hi^	5.3 ± 0.9*	3.0 ± 0.3	4.1 ± 0.5	2.7 ± 0.2	3.2 ± 0.4	2.2 ± 0.4
Monocytes^lo^	3.5 ± 0.4	2.9 ± 0.2	4.9 ± 0.4	4.6 ± 0.3	4.0 ± 0.2	3.6 ± 0.3
B cells	14.1 ± 1.2*	10.7 ± 0.5*	3.5 ± 0.7	2.6 ± 0.6	15.1 ± 1.3*	13.5 ± 1.4*
T cells	9.1 ± 1.0	10.5 ± 0.8	18.1 ± 1.3	22.6 ± 1.1	19.6 ± 3.0	19.4 ± 2.0
Cytotoxic	0.3 ± 0.1	0.5 ± 0.2	0.3 ± 0.1	0.2 ± 0.1	24.9 ± 1.8*	23.5 ± 1.3*
T helper cells	0.8 ± 0.3	0.9 ± 0.3	50.0 ± 5.2*	44.7 ± 7.3*	31.2 ± 4.7*	44.7 ± 4.8*
DN T cells	94.8 ± 4.1*	94.7 ± 3.7*	49.7 ± 5.2	54.9 ± 7.3	43.7 ± 4.3	31.5 ± 5.6
Unstained	10.8 ± 2.3	12.1 ± 3.0	22.2 ± 2.9	21.6 ± 3.2	17.2 ± 2.5	12.9 ± 3.3

Values are means ± SE, *n* = 8 animals. Percentage of leukocyte cell subtypes as a proportion of the total amount of immune cells in the kidney is shown. Myeloid and T cell subtypes are expressed as a percentage of the parent population (myeloid cells and T cells). Unstained leukocytes are expressed as a percentage of live leukocytes. DN T cells, double-negative T cells.

* Significantly (*P* < 0.05) different between protocols; #significantly (*P* < 0.05) different between sex (two-way ANOVA with Šidák post hoc analysis).

**Figure 9.**
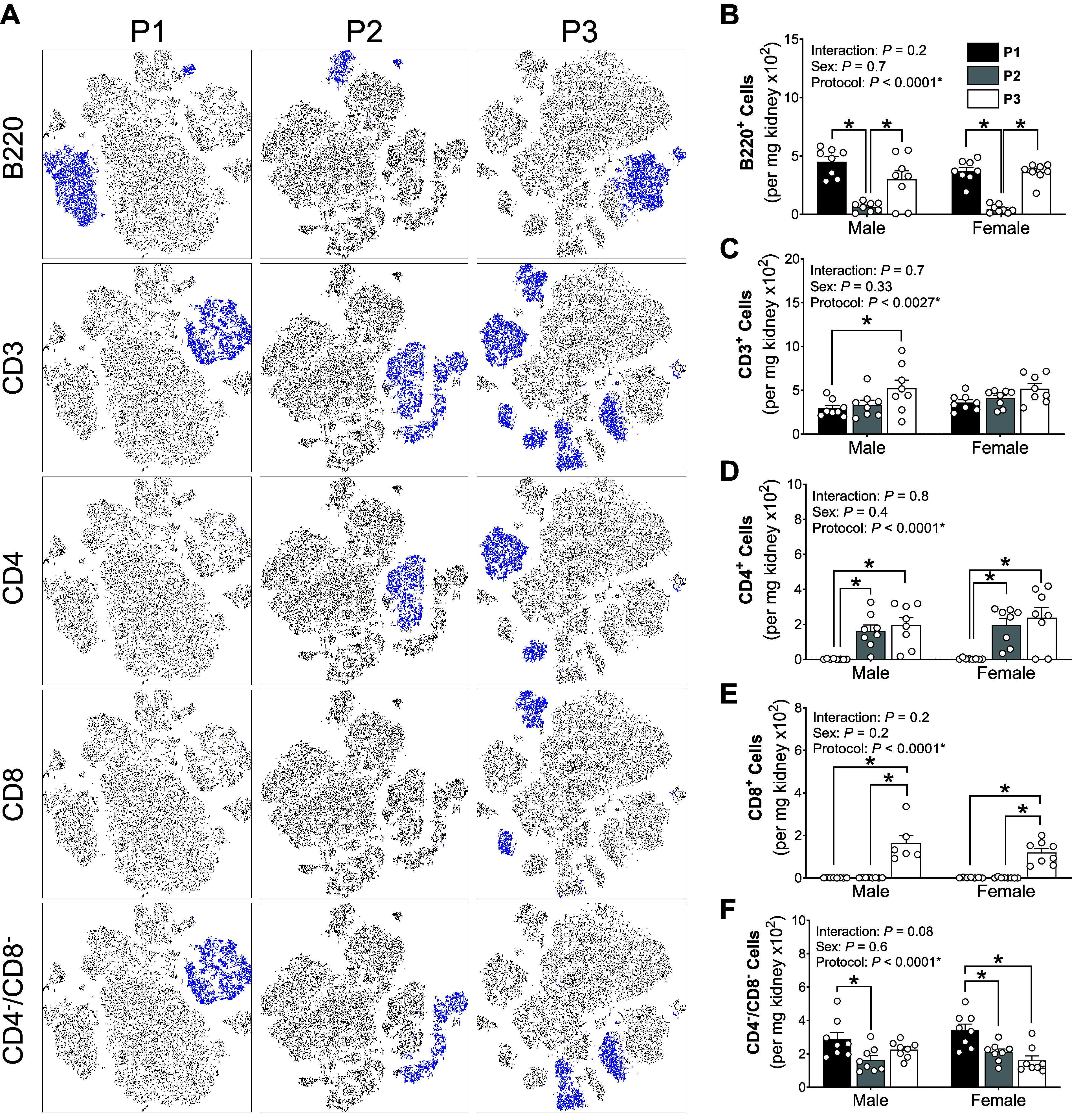
*Protocol 2* single-cell preparation protocol reduced lymphocytes. *A*: representative t-SNE plots of kidney samples process using *protocols 1*,* 2*, and* 3* (*P1*, *P2*, and *P3*, respectively). Four kidney samples were concatenated from each protocol where blue dots depict cell that express the defined marker (*left*). *B−F*: total B cells (B220^+^; *B*), T cells (CD3^+^; *C*), T helper and regulatory T cells (CD4^+^; *D*), cytotoxic T cells (CD8^+^; *E*), and double negative T cells (CD4^−^/CD8^−^; *F*) in male and female kidneys processed using *P1* (black bars), *P2* (gray bars), and *P3* (white bars) single-cell preparation protocols. *Significantly (*P* < 0.05) different between protocols (two-way ANOVA with Šidák post hoc analysis). Values are means ± SE, *n* = 7 or 8 animals.

B cells (B220^+^) were significantly reduced in *P2* preparations compared with *P1* and *P3* (Fig. 9, *A* and *B*). *P3* significantly increased total T cell yields compared with *P1* in males only (CD3^+^; Fig. 9*C*). We also quantified T cell subtypes in each of the single-cell suspensions. Helper T cells (CD4^+^) were enriched in both *P2* and *P3* compared with *P1* (Fig. 9*D*). Cytotoxic (CD8^+^) T cells were significantly enriched by *P3* compared with *P1* and *P2* (Fig. 9*E*). Conversely, double negative (DN) T cells (CD4^−^/CD8^−^) were significantly enriched in male *P1* preparations compared with that of *P2*. Female double negative T cell *P1* preparations were significantly greater than both *P2* and *P3* (Fig. 9*F*).

**Figure 10.**
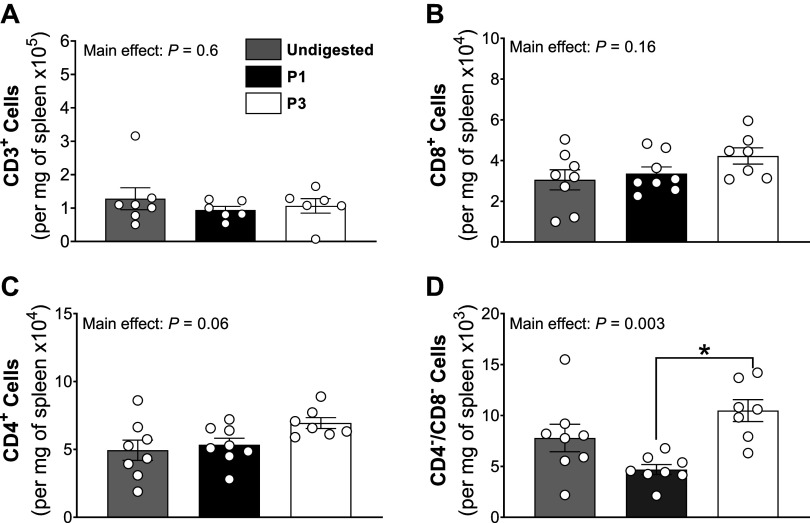
Replication of single-cell preparation *protocols 1* and *3* in the spleen. *A−D*: T cell (CD3^+^; *A*), T helper cell (CD4^+^; *B*), cytotoxic T cell (CD8^+^; *C*), and double-negative T cell (CD4^-^/CD8^-^; *D*) counts were quantified per mg of spleen tissue from female mice via flow cytometry using three digestion methods: undigested (gray bars), *protocol 1* (black bars), and *protocol 3* (white bars). *Significantly (*P* < 0.05) different between protocols (two-way ANOVA with Šidák post hoc analysis). Values are means ± SE, *n* = 6–8 animals.

To provide insights into whether digestion protocols altered absolute cell counts or degraded CD4 and/or CD8 antigens, we prepared single-cell digestions of the spleen using *P1*, *P3*, and red blood cell lysis alone (referred to as “undigested”). However, these enzymatic digestion in spleen did not replicate the results seen in kidney preparations. There were no significant effects of digestion protocol on total T cell, cytotoxic T cell, or T helper cell populations (Fig. 10, *A–C*). However, *P1* significantly decreased double negative T cells compared with *P3* (Fig. 10*D*). These findings suggest that the differences observed in the kidney preparations were due to changes in cell number, rather than CD4/CD8 antigen degradation.

### Immunofluorescence Staining of the Mouse Kidney

**Figure 11.**
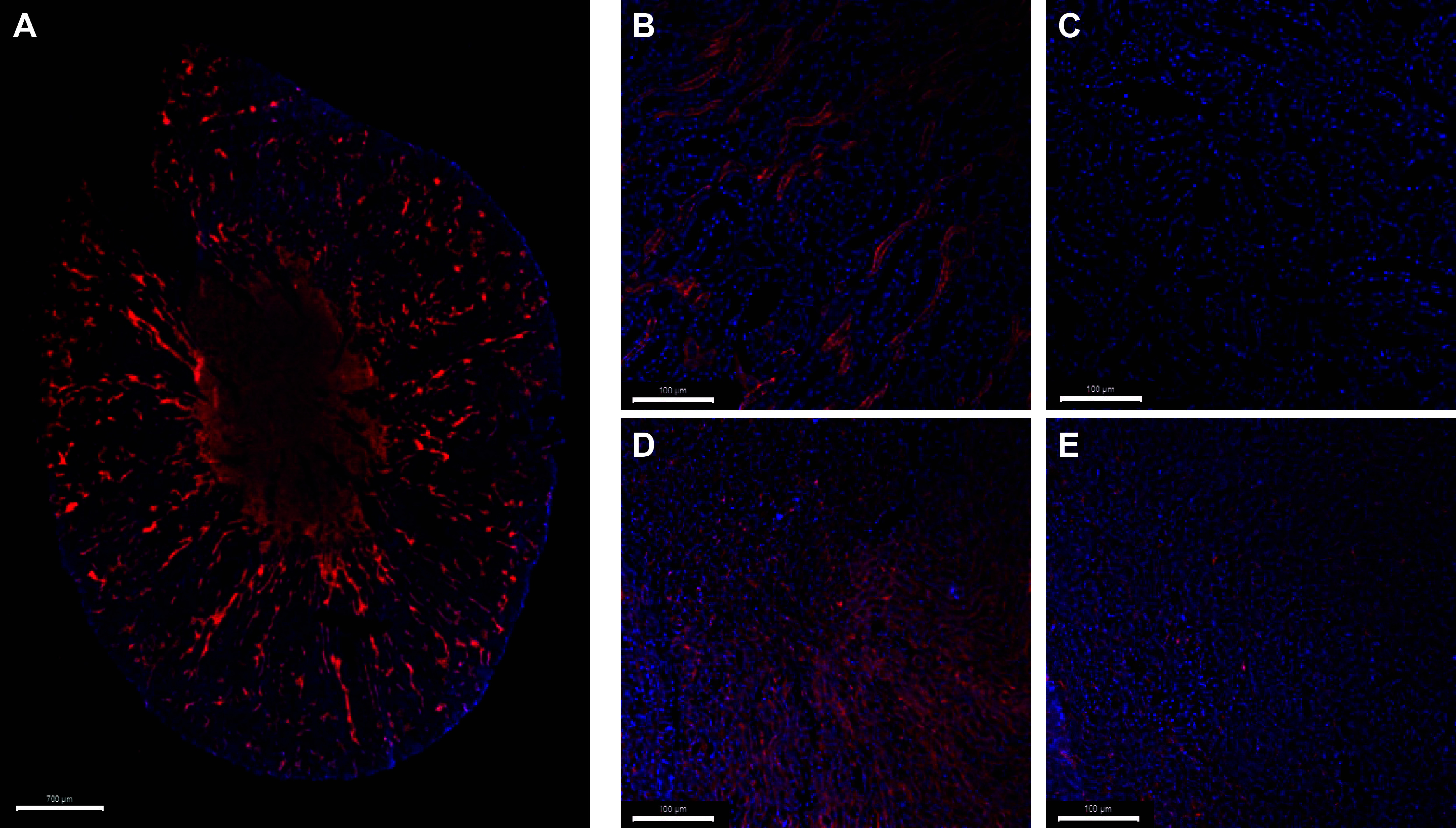
Representative micrographs of immunofluorescent staining of epithelial cell adhesion molecule (EpCAM) in the mouse kidney. *A*: ×20 magnification, tiled image of EpCAM^+^ (epithelial cells) immunofluorescent staining, depicted in red (Cy3). *B*: staining of the renal cortex at ×20 magnification (EpCAM^+^/DAPI^+^). *C*: control sample of the renal cortex imaged at ×20 (EpCAM^−^/DAPI^+^). *D*: EpCAM staining of the renal medulla at ×20 magnification observed in red (EpCAM^+^/DAPI^+^). *E*: control sample of the renal medulla at ×20 magnification (EpCAM^−^/DAPI^+^).

Due to the relatively low abundance of epithelial cells identified through flow cytometry, we validated the EpCAM antibody binding to epithelial cells using immunofluorescence (Fig. 11). The proportion of kidney cells with EpCAM staining was notably greater than the proportions shown in flow cytometry preparations (∼1–5% of all live cells). EpCAM staining was most abundant in the medulla but also present in the cortex. EpCAM staining was not detected in the negative control. Overall, the immunofluorescence experiments suggest that the EpCAM antigen may have been degraded during the digestion protocols, or that the staining protocol used for flow cytometry was not optimal for the EpCAM antibody.

## DISCUSSION

This study is the first to compare the renal cellular yields of three single-cell digestion protocols using high-definition flow cytometry. We found that commonly used debris clearance almost completely removes renal epithelial cells. *P1* enriched both endothelial and myeloid-derived cells while diminishing renal T cell subtypes. *P3* increased epithelial and T cell yields. Notably, *P2*, the most published method for renal single-cell studies, did not enrich any of the cell types studied and produced considerably lower yields for leukocytes (particularly B cells and cytotoxic T cells). Our data also identified small but significant sex differences in renal single-cell preparations. Specifically, granulocytes were increased in male mice whereas smooth muscle cells were increased in female mice. By identifying which digestion protocols produce the best yields for certain cell types, our findings provide important new information that can guide the experimental design of future renal single-cell studies.

Debris clearance is frequently used when single-cell suspensions are prepared from tissues with a rich extracellular matrix—such as the kidney (17). Although this step can improve the proportion of live cells within single-cell preparations, we found that debris clearance dramatically reduced epithelial cell yields in kidney samples. Considering that epithelial cells are a major cell-type in the kidney, this step was removed from subsequent experiments. The debris clearance process may have eliminated epithelial cells in two ways. Epithelial cells may have remained in the discarded supernatant due to their relatively smaller size compared with other cells such as endothelial cells and leukocytes (30, 31). Alternatively, epithelial cell loss may have occurred via increased agitation caused by the debris clearance step. Digestion protocols that cause increased cellular agitation can preferentially reduce the detection of certain cell populations such as epithelial cells, in flow cytometry, due to mechanical breakdown of the epitopes (such as EpCAM) or potentially cause cell rupture (32). The use of other epithelial-cell specific markers (e.g., AE3, CK4) may have identified that debris clearance did not remove epithelial cells, but rather cleaved or removed the EpCAM epitope from cells.

One unexpected finding was the high yield of vascular smooth muscle cells (VSMCs) following a combination of GentleMACs dissociation and *P2* digestion. Notably, no other cell type benefited from this combination and GentleMACS dissociation decreased the abundance of most other cell types when compared with fine mincing with dissection scissors regardless of the single-cell digestion preparation.

One of the most striking differences between the three digestion protocols was the enrichment of endothelial and myeloid-derived cell types (specifically macrophages and proinflammatory monocytes) in *P1*. This protocol has previously shown high endothelial cell and macrophage yields in both the heart and kidney for scRNAseq studies (19, 33, 34). *P1* may be useful for studies that intend to focus on endothelial cells or macrophages. Higher cell proportions or yields of a specific cell type can aid in characterizing cellular heterogeneity within that population. However, *P1* may also lead to the imbalanced sequencing depth and relative representation of certain cell types. Thus, it is important that studies using this technique recognize this potential limitation when considering cellular proportions in the kidney. Furthermore, it is vital to control for overabundant cell populations particularly for scRNAseq studies since sequencing depth will be imbalanced toward the more abundant cell types, limiting the information that can be obtained from minor cell populations (34).

Another interesting finding from *P1* was the relatively low abundance of T cell subtypes (CD4^+^ and CD8^+^) compared with *P2* and *P3*. Due to the relatively high abundance of double negative T cells in *P1* preparations, we suspected that CD4^+^ and CD8^+^ T cells were reduced due to the enzymatic cleavage of these epitopes. However, when comparing *P1* to an undigested solution (red blood cell lysis alone) in the spleen, CD4^+^ and CD8^+^ T cell populations were comparable. This suggests that the low abundance of CD4^+^ and CD8^+^ T cells in renal *P1* preparations was possibly due to the absence of these cell types, rather than epitope cleavage. This is further supported by findings from a scRNAseq study that used *P1* to prepare cardiac single-cell suspensions. Although T cells were subclustered into two subtypes corresponding to CD4^+^ and CD8^+^ T cells, the gene expression of *Cd4* and *Cd8a* was not expressed in all cells within each respective subcluster. Considering that double negative T cells were not identified in this scRNAseq study, it is possible that many of the cells in the two T cell subclusters were in fact double negative T cells (33). Although, it is important to note that *Cd4* and *Cd8a* expression may have indeed been expressed in these cells however, the sequencing reads were not deep enough to detect it. One limitation of this study is the large amount of DN T cells found in all three single-cell digestion preparations. Typically, ∼25% of kidney T cells are DN compared with the ∼30–95% observed in this study (35). Considering that the majority of γδ T cells are thought to be DN, and that γδ T cells play significant roles in various renal pathologies, one limitation of our study is not including a γδ T cell marker in the immune cell panel.

*P2* produced lower leukocyte yields than the other two digestion methods—likely due to a reduction in B cell yields. Previous renal scRNAseq studies using *P2* have notably scarce B cell populations (4, 36). Moreover, a renal scRNAseq study using *P1* had a much more sizeable B cell population in its data set (19). Overall, this indicates that *P2* is not ideal for single-cell studies that wish to investigate B cell biology in renal tissues. Although *P2* produced the lowest yields for many cell types, this digestion method is the most widely used in kidney scRNAseq studies (4, 6, 36). This highlights the importance of research such as that of the current study to optimize cellular yields for single-cell sciences. More refined single-cell suspensions will increase the accuracy and relevance of data interpretations, which will facilitate better detection of novel targets for the intervention of CKD at early stages of development (5, 6).

Overall, the single-cell preparation protocols worked similarly between sexes. However, our study did identify some novel differences between renal cell populations that were independent of protocol. Renal granulocyte populations were demonstrated to be significantly greater in male mice when compared with females. This difference is likely due to neutrophils and as previous research shows that non-neutrophil granulocytes are not significantly different between men and women (37). Other research has shown that neutrophils are significantly greater in male blood samples compared with women (38). Currently, there is a lack of data investigating sexual dimorphisms in mouse renal cells. This makes speculation into the cause of these differences difficult and highlights a need for increased research into sexual differences. Another renal cell type that demonstrated sexual dimorphisms was vascular smooth muscle cells (VSMCs). VSMCs were significantly greater in female mice compared with males. We hypothesize this may have been due to increased vascularity in female kidneys that has been shown to be modulated by estrogen in a study (39). The G protein-coupled estrogen receptor has been demonstrated to be present in renal cells and its activation in female mice improves the survivability of VSMCs in kidney disease models. This may increase the baseline amount of VSMCs in female mice, however, this would need to be investigated further to ameliorate the genetic pathways involved.

A low abundance of epithelial cells was observed in all preparations. Most live cells within these preparations were unbound to a fluorescent antibody (Table 4). Epithelial cells are the most abundant cell type in the kidney and therefore, we can assume that most of these “unbound” cells were indeed epithelial (4, 5). This suggests that the EpCAM epitope was particularly susceptible to cleavage during the process of digestion by enzymes or that the staining conditions used for this experiment were not optimal (staining protocol followed manufacturer’s instructions). This hypothesis is supported by the immunofluorescence staining used with the same EpCAM antibody (Fig. 5). Immunofluorescent staining showed that EpCAM is not ubiquitous throughout the renal epithelia. However, a greater proportion of cells showed fluorescent staining than when comparing with the data from flow cytometry. This suggests that indeed EpCAM is particularly susceptible to enzymatic cleavage and may not serve as an optimal marker for the renal epithelia during studies using flow cytometry.

The three single-cell digestion preparations exhibited significant differences in kidney cellular heterogeneity. These findings highlight the importance of the digestion protocol when planning a single-cell science study. The single-cell digestion has the potential to shift the cellular environment such that particular populations can be enriched in the single-cell suspension. Downstream this could result in a loss of cells of interest for techniques like flow cytometry or a shift in the genetic landscape for techniques like scRNAseq. The findings in this study may help future single-cell science research and guide researchers to choose specific protocols that may enrich for cell types of interest.

## DATA AVAILABILITY

Data will be made available upon reasonable request.

## GRANTS

This work was funded by Jack Brockhoff Foundation Fellowship ID4519 (to M.J.), a Diabetes Australia Research Program General Grant (to M.J., A.V., and G.R.D.), and NHMRC Ideas Grant GNT2020452 (to G.R.D., A.V., and M.J.). M.J. was supported by joint NHMRC and NHF Postdoctoral Fellowships GNT1146314 and 101943. J.N.R. was supported by an Australian Research Training Scholarship and a Defence Science Institute Research Higher Degree Student Grant.

## DISCLOSURES

No conflicts of interest, financial or otherwise, are declared by the authors.

## AUTHOR CONTRIBUTIONS

J.N.R., A.R.P., G.R.D., A.V., and M.J. conceived and designed research; J.N.R., H.D., A.V., and M.J. performed experiments; J.N.R., A.V., and M.J. analyzed data; J.N.R., G.R.D., A.V., and M.J. interpreted results of experiments; J.N.R., A.V., and M.J. prepared figures; J.N.R., A.V., and M.J. drafted manuscript; J.N.R., H.D., A.R.P., C.G.S., G.R.D., A.V., and M.J. edited and revised manuscript; J.N.R., H.D., A.R.P., C.G.S., G.R.D., A.V., and M.J. approved final version of manuscript.
